# Supplementary material for: Shigella flexneri evades LPS ubiquitylation through IpaH1.4-mediated degradation of RNF213
Source: Nat Struct Mol Biol. 2025 Apr 9;32(9):1741–51. doi: 10.1038/s41594-025-01530-8 (PMC12440826; doi:10.1038/s41594-025-01530-8)
Supplement: Supplementary file 1 — Reporting Summary [file 41594_2025_1530_MOESM1_ESM.pdf]

nature portfolio

Corresponding author(s): Felix Randow

Last updated by author(s): Jan 31, 2025

Reporting Summary

Nature Portfolio wishes to improve the reproducibility of the work that we publish. This form provides structure for consistency and transparency in reporting. For further information on Nature Portfolio policies, see our Editorial Policies and the Editorial Policy Checklist.

Statistics

For all statistical analyses, confirm that the following items are present in the figure legend, table legend, main text, or Methods section.

n/a	Confirmed	
☐	☒	The exact sample size (*n*) for each experimental group/condition, given as a discrete number and unit of measurement
☐	☒	A statement on whether measurements were taken from distinct samples or whether the same sample was measured repeatedly
☐	☒	The statistical test(s) used AND whether they are one- or two-sided<br>*Only common tests should be described solely by name; describe more complex techniques in the Methods section.*
☒	☐	A description of all covariates tested
☒	☐	A description of any assumptions or corrections, such as tests of normality and adjustment for multiple comparisons
☐	☒	A full description of the statistical parameters including central tendency (e.g. means) or other basic estimates (e.g. regression coefficient) AND variation (e.g. standard deviation) or associated estimates of uncertainty (e.g. confidence intervals)
☐	☒	For null hypothesis testing, the test statistic (e.g. F, t, r) with confidence intervals, effect sizes, degrees of freedom and P value noted<br>*Give P values as exact values whenever suitable.*
☒	☐	For Bayesian analysis, information on the choice of priors and Markov chain Monte Carlo settings
☒	☐	For hierarchical and complex designs, identification of the appropriate level for tests and full reporting of outcomes
☒	☐	Estimates of effect sizes (e.g. Cohen's *d*, Pearson's *r*), indicating how they were calculated

Our web collection on statistics for biologists contains articles on many of the points above.

Software and code

Policy information about availability of computer code

Data collection: Commercial software: EPU v3.7 (cryoEM data collection); Digital Micrograph v3; Zeiss ZEN (fluorescence microscopy imaging); BioRad Image Lab Touch v2.4 (imaging of blots and gels); UNICORN v5.0 (AKTA for protein purification); BD FACS Diva v9.0 (flow cytometry).
No custom software.

Data analysis: Open source and commercial software: RELION v5.0, CTFFIND v4.1, Resmap v1.1, cryoEF v1.2, pyEM v0.4, cryoSPARC v4.2-4.3 (cryoEM data analysis); ChimeraX v1.7-1.8, ISOLDE v1.6, coot v 0.9, Phenix v1.17 (atmoic model building, refinement, and visualization); FIJI (fluorescence image display); GraphPad Prism v10, Anaconda v3 for Python v3.6 (graphs); FlowJo v9-10 (flow cytometry).
No custom software.

For manuscripts utilizing custom algorithms or software that are central to the research but not yet described in published literature, software must be made available to editors and reviewers. We strongly encourage code deposition in a community repository (e.g. GitHub). See the Nature Portfolio guidelines for submitting code & software for further information.

Data

Policy information about availability of data

All manuscripts must include a data availability statement. This statement should provide the following information, where applicable:

- Accession codes, unique identifiers, or web links for publicly available datasets
- A description of any restrictions on data availability
- For clinical datasets or third party data, please ensure that the statement adheres to our policy

The cryoEM maps and the refined atomic models have been deposited in Electron Microscopy Data Bank and the Protein Data Bank under the accession codes EMD 50913–50929 and PDB 9G08/9G09, respectively. Other data are available within the article and its associated supplementary information files. The following publicly available atomic models were retrieved from the Protein Data Bank for the purpose of model building or comparison: PDB 7V8G, 7V8F, 7YA7, 7YA8, 8S24.

Research involving human participants, their data, or biological material

Policy information about studies with human participants or human data. See also policy information about sex, gender (identity/presentation), and sexual orientation and race, ethnicity and racism.

Reporting on sex and gender	No human participants involved in the research.
Reporting on race, ethnicity, or other socially relevant groupings	No human participants involved in the research.
Population characteristics	No human participants involved in the research.
Recruitment	No human participants involved in the research.
Ethics oversight	No human participants involved in the research.

Note that full information on the approval of the study protocol must also be provided in the manuscript.

Field-specific reporting

Please select the one below that is the best fit for your research. If you are not sure, read the appropriate sections before making your selection.

☒ Life sciences ☐ Behavioural & social sciences ☐ Ecological, evolutionary & environmental sciences

For a reference copy of the document with all sections, see nature.com/documents/nr-reporting-summary-flat.pdf

Life sciences study design

All studies must disclose on these points even when the disclosure is negative.

Sample size	No statistical methods were used to determine sample size. For cryoEM, the sample size was determined by the data collection time available and was sufficient as indicated by the resolution reached in the refined maps. The numbers of micrographs and particles are listed in Supplementary Table S1. For blots, the sample size (number of cells per sample) was chosen so as to give unambiguous signal in positive control and loading control lanes, and was always greater than 10,000 cells per lane. For flow cytometry and cell counting, the sample sizes are similar to those generally used in the field and are stated in the figure legends; the statistical results from biological replicates show that the sample sizes chosen were sufficient for statistical significance and reproducibility. For flow cytometry, analysing at least 10,000 cells per sample is sufficient to ensure the error on the Poisson event counting is less than or equal to 1%, which is smaller than the typical differences between the analysed samples and the standard deviation between biological replicates. For cell counting the sample size was also sufficient to ensure that the counting error is less than the standard deviation between biological replicates, hence further increase in the sample size was not deemed necessary. Similar sample sizes for counting of infected cells have been previously used by us and others, see e.g. Otten et al, Nature, 2021.
Data exclusions	No technical replicates were excluded. For cryoEM data processing, particles were excluded using standard classification algorithms and those excluded corresponded to contaminants, false picks, or incomplete complexes (as described in Extended Data Figure 7C).
Replication	Experiments shown in the manuscript were reliably reproducible by multiple authors. Number of independent biological repeats are stated in the figure legends.
Randomization	Cells and bacteria from common pools were split randomly for the different experiments. For the cryoEM data analysis, the particles were split into random subsets (mini-batches) or half-sets during 2D/3D classification and refinement.
Blinding	Blinding was not relevant to this study as the experiments were conducted by individual researchers and required keeping note of the identity of the specimens.

Reporting for specific materials, systems and methods

We require information from authors about some types of materials, experimental systems and methods used in many studies. Here, indicate whether each material, system or method listed is relevant to your study. If you are not sure if a list item applies to your research, read the appropriate section before selecting a response.

Materials & experimental systems

n/a	Involved in the study
☐	☒ Antibodies
☐	☒ Eukaryotic cell lines
☒	☐ Palaeontology and archaeology
☒	☐ Animals and other organisms
☒	☐ Clinical data
☒	☐ Dual use research of concern
☒	☐ Plants

Methods

n/a	Involved in the study
☒	☐ ChIP-seq
☐	☒ Flow cytometry
☒	☐ MRI-based neuroimaging

Antibodies

Antibodies used	FK2 (Enzo Life Science, BML-PW8810, against conjugated ubiquitin, 1:1000), anti-GroEL (Enzo Life Science, ADI-SPS-875-F, 1:2000), anti-actin (Abcam, ab8227, 1:1000), anti-RNF213 (Merck, HPA003347 and HPA026790, 1:1000), anti-Flag–M2–HRP (Merck, A8592, 1:1000), anti-GFP (JL8, Clontech, 632381, 1:2000), anti-K48-linked ubiquitin (Abcam, ab140601, 1:1000), anti-Salmonella typhimurium LPS (BioRad, 8210-0407, 1:100), Salmonella O Antisera Group B (BD Difco, 229481, 1:100). Secondary antibodies: AF488-Goat-anti-Rabbit IgG (Invitrogen A11034, 1:500), Goat Anti-Mouse Immunoglobulins/HRP (Dako P0447, 1:5000), Goat Anti-Rabbit Immunoglobulins/HRP (Dako P0448, 1:5000).
Validation	Antibodies were validated by the manufacturers by western blotting as follows: FK2 - on ubiquitin chains (K29, K48, K63-linked); anti-GroEL - on recombinant E. coli GroEL; anti-actin - on HeLa cell lysate; anti-RNF213 - on A549 and HEK293 cell lysate; anti-Flag-M2-HRP - on Flag-transfected COS cells; anti-GFP - on cell lysate from HEK293 stably expressing AcGFP1; anti-K48-linked ubiquitin - on K48-linked-Ub2-7 recombinant protein. Anti-Salmonella typhimurium LPS antibody was validated by the manufacturerusing ELISA and IF against Salmonella typhimurium and negative controls (S. paratyphi A, S. choleraesuis, S. newport, S. enteriditis, S. anatum, S. selandia, E. coli 055:B5, E. coli K12, Klebsiella pneumoniae). Salmonella O Antisera Group B was validated by the manufacturer using the slide agglutination test on a variety of Salmonella cultures. Secondary antibodies were validated by the manufacturer using ELISA with immunoglobulins from various species.

Eukaryotic cell lines

Policy information about cell lines and Sex and Gender in Research

Cell line source(s)	HeLa cells, HEK293T cells and mouse embryonic fibroblasts were obtained from ATCC. The RNF213 KO cell lines have been previously described (Otten et al, Nature, 2021).
Authentication	No authentication was performed.
Mycoplasma contamination	All cells tested negative for mycoplasma.
Commonly misidentified lines (See ICLAC register)	No commonly misidentified cell line was used.

Plants

Seed stocks	No plants involved in the research.
Novel plant genotypes	No plants involved in the research.
Authentication	No plants involved in the research.

Flow Cytometry

Plots

Confirm that:

☒ The axis labels state the marker and fluorochrome used (e.g. CD4-FITC).

☒ The axis scales are clearly visible. Include numbers along axes only for bottom left plot of group (a 'group' is an analysis of identical markers).

☒ All plots are contour plots with outliers or pseudocolor plots.

☒ A numerical value for number of cells or percentage (with statistics) is provided.

Methodology

Sample preparation	Cultured cells, live or fixed in 4% paraformaldehyde, and washed in PBS, as described in the Mehtods section.
Instrument	BD LSRFortessa, BD FACS AriaFusion
Software	Data collection: BD FACS Diva v 9.0. Data analysis: FlowJo v9-10.
Cell population abundance	A total of 10,000 – 30,000 cells per sample were analyzed, as specified in the text. The relevant cell population abundances are quantified in Figures 3A and 4G, as described in Extended Data Figure 3A. The relevant cell population abundances are quantified in Extended Data Figures 4 and 9, as described therein.
Gating strategy	No FSC/SSC gating was applied. Fluorescence +/- boundaries for Figure 3 were defined as shown in Extended Data Figure 3A, based on the controls shown in the upper row of Extended Data Figure 3B. For the experiments shown in Extended Data Figures 4 and 9, the gates are shown for each panel separately.

☒ Tick this box to confirm that a figure exemplifying the gating strategy is provided in the Supplementary Information.
