# Supplementary material for: Shigella flexneri evades LPS ubiquitylation through IpaH1.4-mediated degradation of RNF213
Source: Nat Struct Mol Biol. 2025 Apr 9;32(9):1741–51. doi: 10.1038/s41594-025-01530-8 (PMC12440826; doi:10.1038/s41594-025-01530-8)

Figure 1B

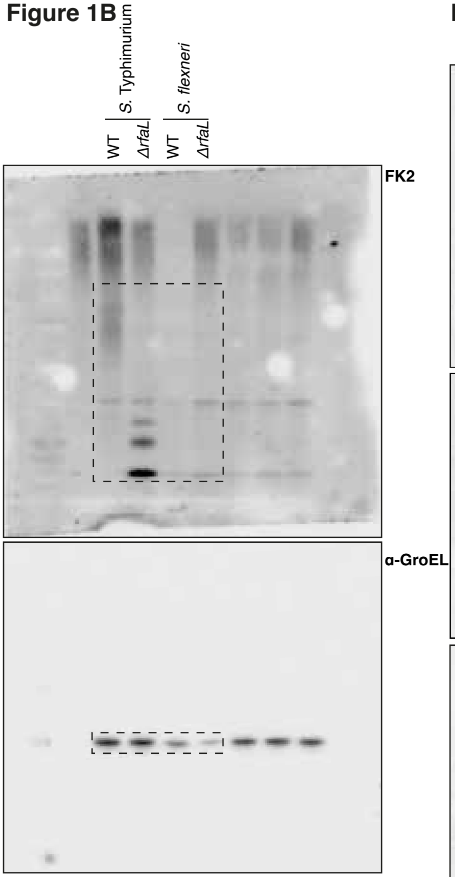

Figure 2A

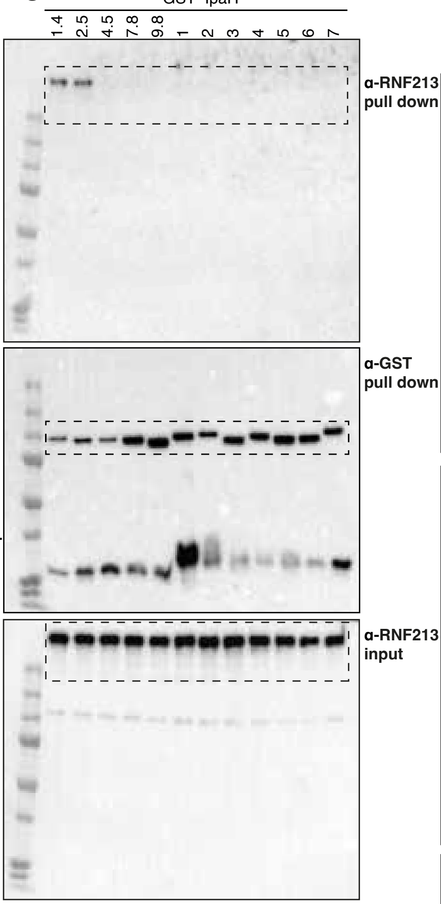

Figure 2B (ct'd)

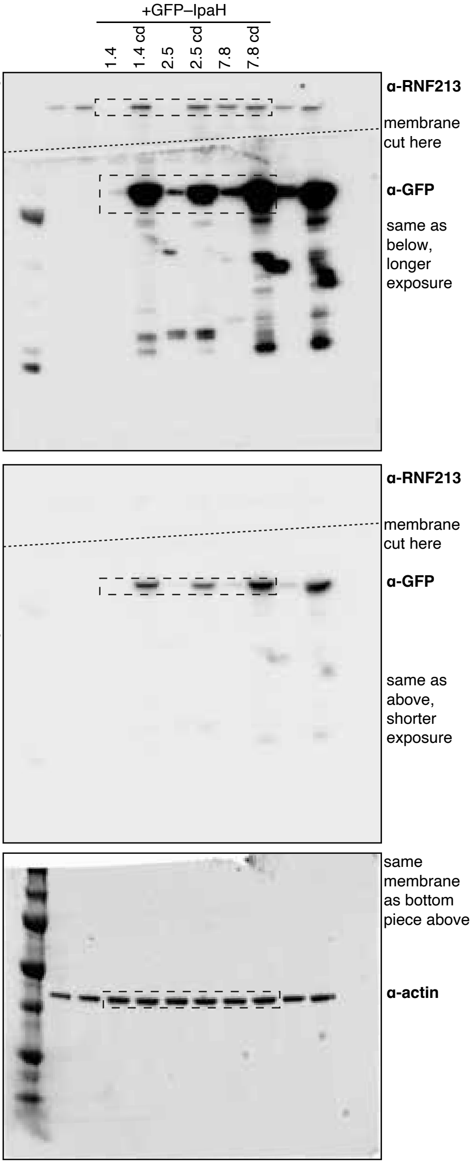

Figure 2C

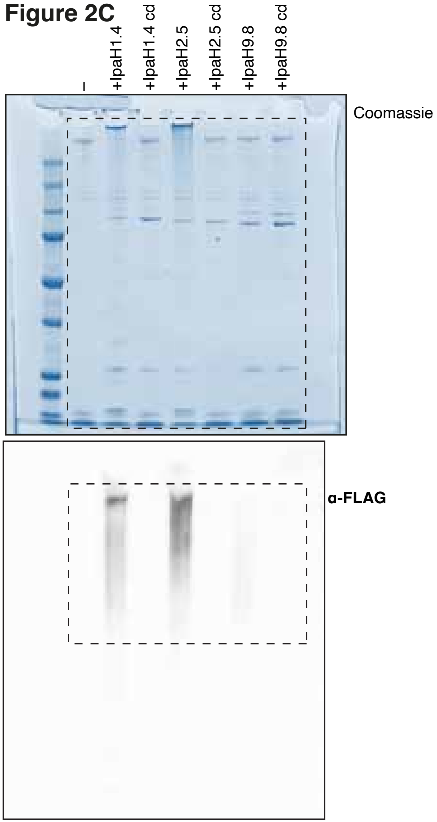

Figure 2B

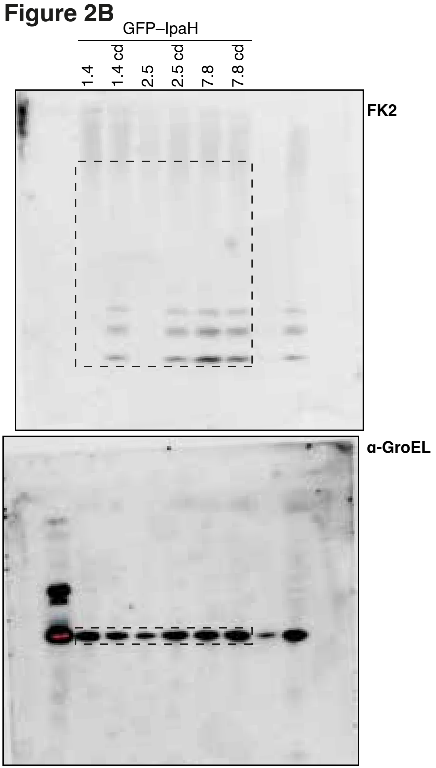

Figure 3C & Extended Data Figure 5C

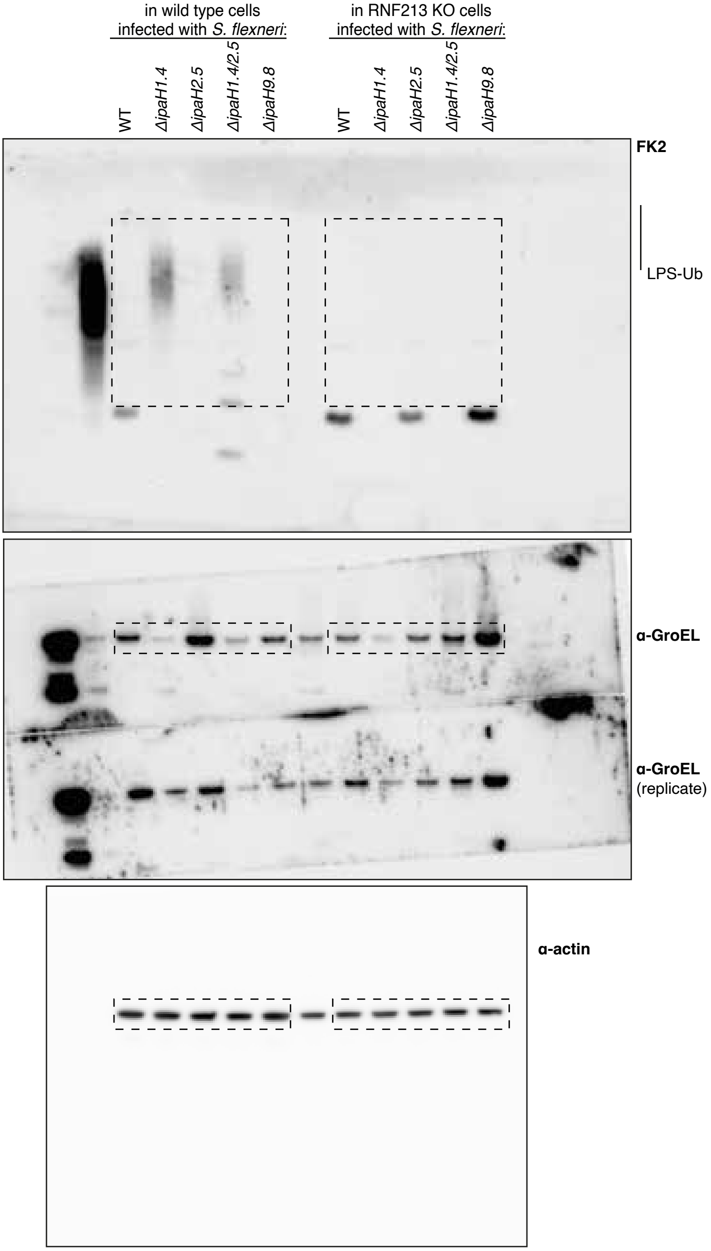

Extended Data Figure 2A

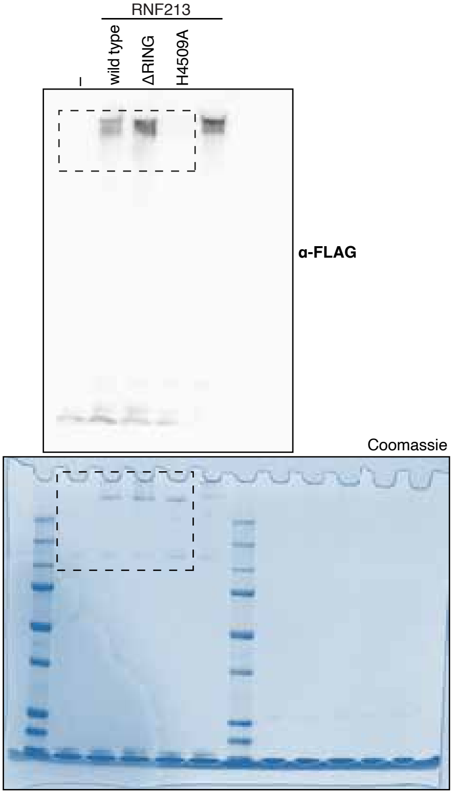

Extended Data Figure 2B

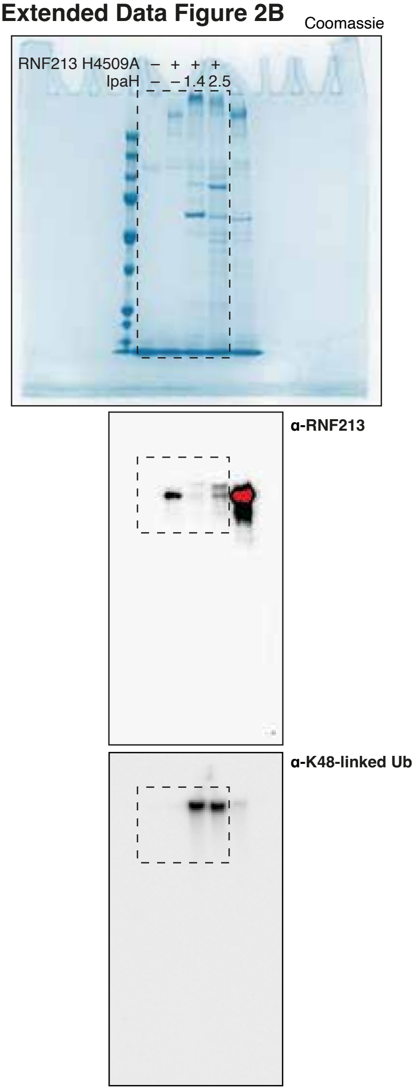

Extended Data Figure 2C

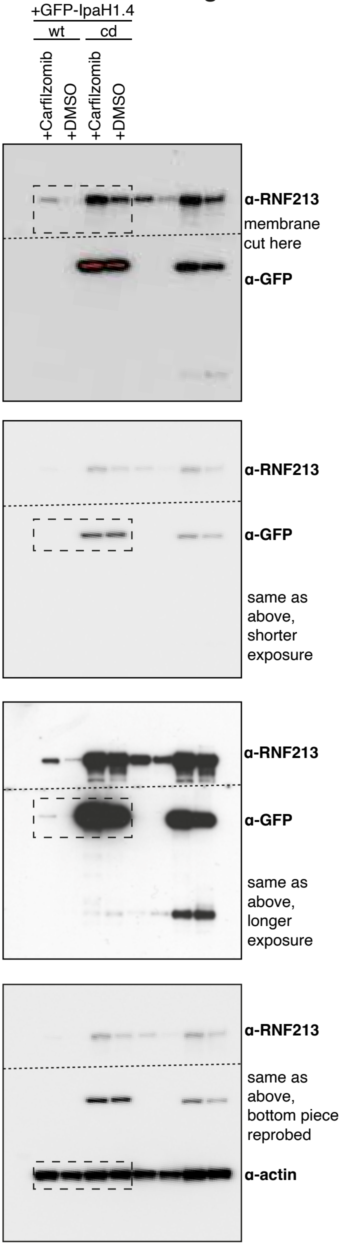

Extended Data Figure 4C

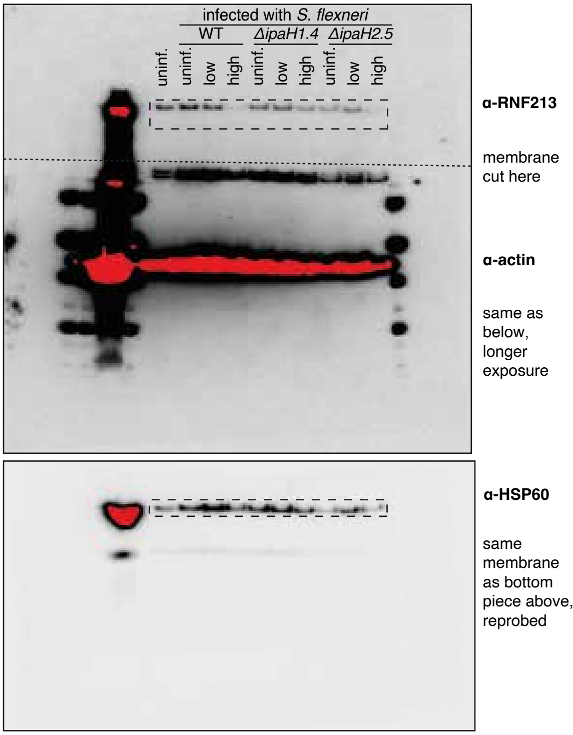

Extended Data Figure 8A

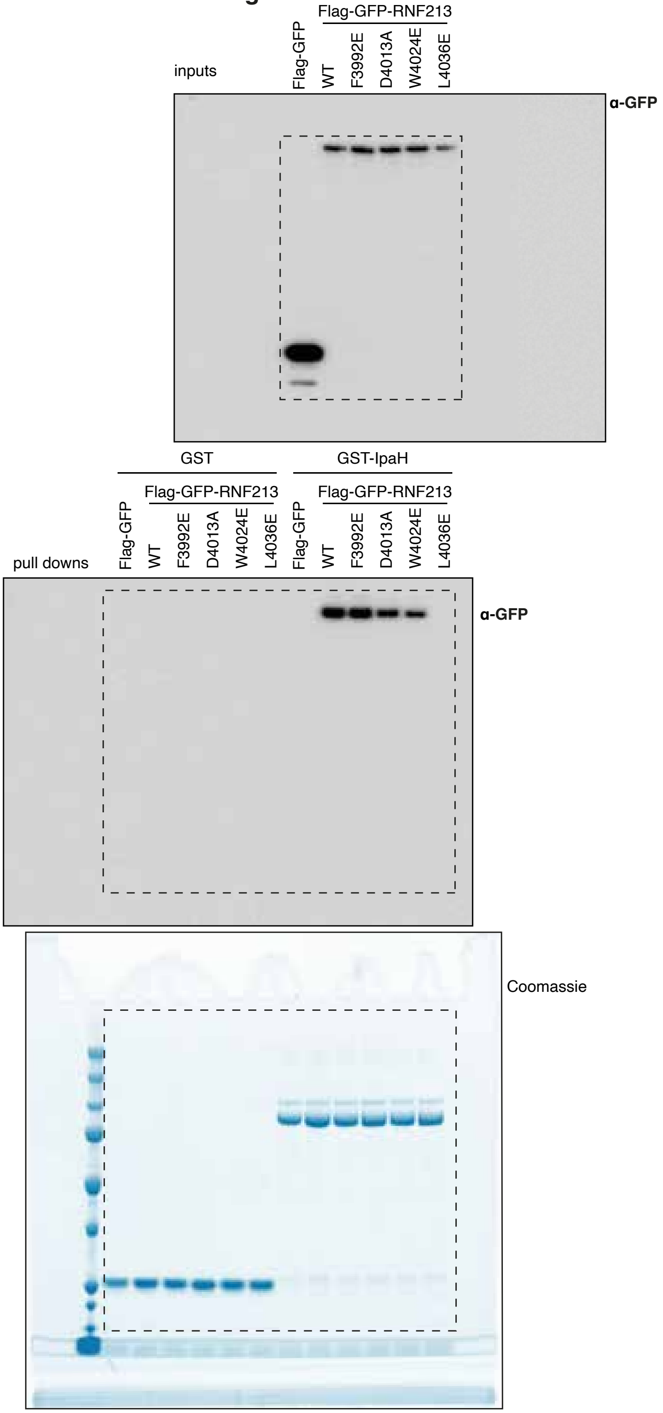

Extended Data Figure 8B

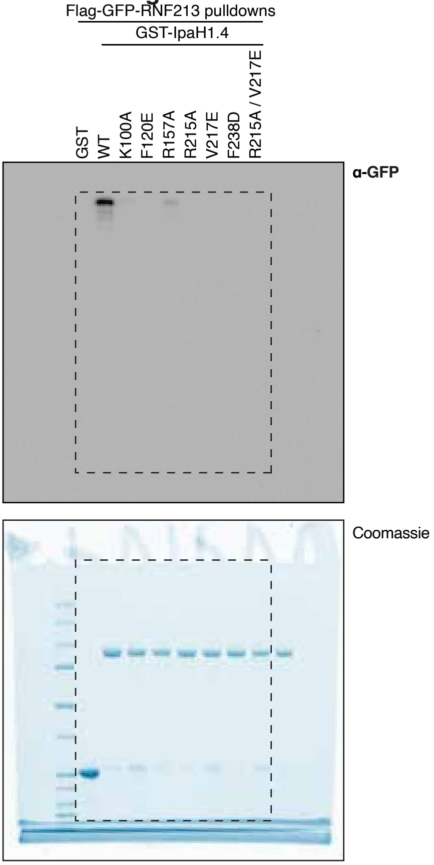

Extended Data Figure 9

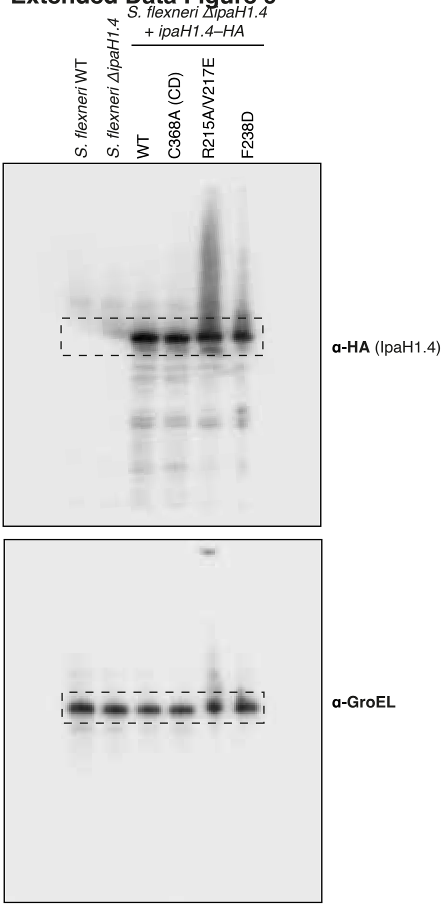

Supplement: Supplementary file 7 — Source data for cropped immunoblots and gels and original images of the cropped immunoblots and gels. [file 41594_2025_1530_MOESM7_ESM.pdf]
